# Primary care patients in psychiatric clinical trials: a pilot study using videoconferencing

**DOI:** 10.1186/1744-859X-6-24

**Published:** 2007-10-04

**Authors:** Janet BW Williams, Amy Ellis, Arthur Middleton, Kenneth A Kobak

**Affiliations:** 1 MedAvante, Inc., MedAvante Research Institute, Hamilton, NJ, USA; 2 Columbia University, Dept. of Psychiatry, New York, NY, USA; 3 Hackensack University Medical Center, Hackensack, NJ, USA

## Abstract

**Background:**

While primary care physicians play a pivotal role in the treatment of depression, collaboration between primary care and psychiatry in clinical research has been limited. Primary care settings provide unique opportunities to improve the methodology of psychiatric clinical trials, by providing more generalizable and less treatment-resistant patients. We examined the feasibility of identifying, recruiting, screening and assessing primary care patients for psychiatric clinical trials using high-quality videoconferencing in a mock clinical trial.

**Methods:**

1329 patients at two primary care clinics completed a self-report questionnaire. Those screening positive for major depression, panic, or generalized anxiety were given a diagnostic interview via videoconference. Those eligible were provided treatment as usual by their primary care physician, and had 6 weekly assessments by the off-site clinician via videoconferencing.

**Results:**

45 patients were enrolled over 22 weeks, with 36 (80%) completing the six-week study with no more than two missed appointments. All diagnostic groups improved significantly; 94% reported they would participate again, 87% would recommend participation to others, 96% felt comfortable communicating via videoconference, and 94% were able to satisfactorily communicate their feelings via video.

**Conclusion:**

Results showed that primary care patients will enroll, participate in and complete psychiatric research protocols using remote interviews conducted via videoconference.

## Background

Primary care physicians have long been recognized as playing a pivotal role in the treatment of depression, providing the majority of all mental health treatments for this disorder [1,2]. They are often the first point of contact for patients with mental health concerns. It has been estimated that up to 22% of primary care patients have some form of co-morbid depression [3,4]. A collaborative model between primary care and mental health specialists that includes such elements as evidence-based treatment protocols, improved methods of screening and detection, patient education, and active monitoring of treatment adherence and outcome, has been shown to be an effective treatment intervention strategy[5-8].

While there has been increased focus on the collaboration between primary care and mental health practitioners in the clinical care of patients, less attention has been paid to collaboration between the two disciplines in clinical research. Primary care settings provide unique opportunities to improve the methodology of clinical trials. Traditionally, clinical trials in depression primarily recruit through newspaper ads, resulting in a high proportion of treatment-resistant and atypical patients. These patients are often more likely to fail treatment, increasing the chances of a failed trial. Primary care patients are more likely to be treatment naïve, as depression is often first identified in a primary care setting. In addition, patients treated in a primary care setting may be less likely to be lost to follow-up, as they usually already have an ongoing relationship with the treatment provider. There is some evidence that primary care patients may also be less likely to respond to placebo: in a series of four generalized anxiety disorder studies, a 20% higher placebo response rate was found in patients recruited from psychiatric settings as compared to primary care sites [9]. The high prevalence rate of depression and other mental health disorders in primary care can also help facilitate patient recruitment for clinical trials.

An obstacle to participation in clinical depression trials by primary care physicians is the lack of formal training in the diagnosis and assessment of depression using standardized rating scales. Other barriers include a lack of experience in research methodology, and limited time for clinical trial management. One potential solution to this problem is the use of off-site expert, centralized raters that are linked to the various study sites through videoconferencing or teleconferencing [10]. These raters can remotely administer the primary outcome measures to study patients during their regularly scheduled study visits. Centralized raters have several advantages, including improved reliability and quality, more thorough calibration and monitoring for interview quality, and reduced rating bias (as they are independent from the study site). Centralized raters can also be blinded to study visit, study design, and study entrance requirements, further reducing expectation biases. Several studies have shown that rating scales administered remotely by videoconference or teleconference yield equivalent results as when administered face-to-face [11-20].

The current study examined the feasibility of identifying, recruiting and screening primary care patients for clinical trials, and examined patient comfort, satisfaction, and adherence in a mock clinical trial using high-quality videoconferencing for screening and ongoing evaluations by remote raters.

## Methods

Patients at two large research-naïve primary care practices were provided a letter at check-in from their primary care physician inviting them to participate in a study of the usefulness of telemedicine in evaluating patient symptoms. Those who were interested in participating completed a self-report questionnaire (the Patient Health Questionnaire; PHQ) [21] in the waiting room to assess their eligibility. The PHQ screens for common mental health disorders found in primary care patients. Those screening positive for major depressive disorder (MDD), generalized anxiety disorder (GAD), or panic disorder (PD) on the PHQ and who were otherwise eligible to participate (i.e. not currently receiving mental health treatment such as psychotherapy or pharmacotherapy, not currently abusing alcohol or drugs, or having suicidal ideation) were scheduled for a diagnostic interview conducted remotely by a well-trained rater using high-quality videoconferencing. The diagnostic interview contained the overview from the Structured Clinical Interview for DSM-IV (SCID) [22] and diagnostic questions from the PHQ. Patients whose positive screen was confirmed by the diagnostic interview were also administered a symptom rating scale: either the Hamilton Depression Rating Scale (HAMD) [23], Hamilton Anxiety Rating Scale (HAMA) [24], or Panic Disorder Severity Scale (PDSS) [25], depending on their primary diagnosis (MDD, GAD, or PD respectively). Structured interview guides were used to administer all rating scales [25-27]. Patients were asked to come back once a week for 6 weeks for follow-up evaluations, as if they were in a therapeutic clinical trial. At their weekly visits, patients were evaluated remotely with either the HAMD, HAMA, or PDSS, as well as the Clinical Global Impressions of Change Scale (CGI-C) [28].

The results of the initial diagnostic interview were shared with the patient's primary care physician. The physician decided, based on this information and his or her knowledge of the patient, whether treatment with medication should be offered or whether another intervention or referral was indicated. Regardless of whether the patient was offered or accepted a medication treatment plan, the patient was invited to participate in the longitudinal tracking phase.

Throughout the study, the data regarding treatment response was available to the treating physician. If the patient's condition deteriorated significantly, the interviewer notified the primary care physician, who followed agreed-upon next steps. A 25% increase in the HAM-D, HAM-A, PDSS score, suicidal ideation or any condition requiring hospitalization mandated withdrawal from the study and immediate treatment (for those not already receiving treatment). At the end of the study, a copy of the research evaluations was made for inclusion in the patient's medical chart.

Patients completed a survey after their first and last remote assessment in which they were asked to rate their overall preferences for the videoconferencing process, their level of agreement with descriptions of various attributes of their experience, and to respond to three open-ended questions probing attitudes towards remote assessment.

The study was approved by the New York State Psychiatric Institute Institutional Review Board, and all patients signed informed consent statements. Patients received $100 for their participation. Patients who discontinued early were paid on a pro-rata basis for number of visits completed.

### Telemedicine equipment and procedure

Remote interviews were conducted using H.323 IP standards-based Polycom iPower Videoconferencing Systems (Polycom, Inc., Pleasanton, CA), connected using ISDN lines (i.e., multiple dedicated phone lines that can handle voice, video and data). The system ran at an industry standard bit rate of 384 kps, was HIPAA compliant, and occurred via a secure encrypted connection. Interviews were conducted in a private room with a desk and a door for privacy. The on-site research coordinator oriented the patient on what to do and where to go when the interview was completed, and answered any questions the patient had. The research coordinator waited with the patient to answer the call from the remote interviewer, then left the room and closed the door to ensure the patient's complete privacy.

## Results

### Study flow

A total of 1329 patients were screened in the waiting room with the PHQ. Of these, 211 patients (16%) screened positive for one of the three mental health disorders on the PHQ. Of these 211, 76 (36%) met one of the exclusion criteria and 56 (27%) declined consent, leaving 79 (37%) patients eligible for participation in the study. Of the 79 consenting patients, 9 were lost to follow-up between consent and the initial remote assessment, leaving 70 subjects who were administered the initial follow-up diagnostic interview by the remote clinician. Of the 70 patients who were available for follow-up, 15 were found to have only subthreshold diagnoses, and 10 were found to have met other exclusionary criteria, leaving 45 patients eligible for the tracking phase of the study.

Enrollment by diagnosis and study site is presented in Table 1. Of the 45 patients enrolled, 17 (38%) had MDD as the primary diagnosis, 14 (31%) had GAD, and 14 (31%) had panic disorder. The proportion of GAD patients vs PD patients in the GAD/PD arm was naturalistic and no attempt to balance to a pre-determined level was made.

**Table 1:** Patient enrollment by diagnosis and study site

**Study arm**	**Site I**	**Site II**	**Total study**
Enrollment target	10	30	
Actual enrollment	12	33	45 (100%)
Major depression	7	10	17 (38%)
Generalized anxiety	1	13	14 (31%)
Panic	4	10	14 (31%)
Total	12	33	45 (100%)

The total time taken to enroll these 45 eligible patients was 22 weeks. Site 1 had a target enrollment of 10 patients, and enrolled 12 patients over 8.4 weeks, and site 2 had a target enrollment of 30 patients, and enrolled 33 patients over 16.6 weeks. There was a 3-week overlap of enrollment time between the sites. Given the staggered enrollment period, the total enrollment rate was approximately two eligible patients per week.

### Study adherence

Of the 45 patients enrolled, 36 (80%) completed the six-week study with no more than two missed appointments. A total of 27 patients (60%) completed the entire series of six visits, 7 patients (16%) completed with one missed interview, 2 patients (4%) completed with two missed interviews, and 9 patients (20%) dropped out.

Reasons for the 9 drop-outs were: patient did not return call (4), phone disconnected (1), parent developed cancer (1), too many medical problems (1), looking for therapy ('not getting anything out of assessments') (1), and patient denied being depressed (1).

There were no significant differences between those completing at least 80% of visits, and those who did not in terms of baseline symptom severity or gender; however, those who completed were significantly older than those who did not (47 vs 34 years, p = 0.022). In addition, a greater percentage of completers (17 of 24) than non-completers (3 of 9) agreed with the statement 'I would prefer to be interviewed in my doctors office using this technology than have to travel to be interviewed by someone face-to-face', χ^2^(1) = 3.855, p = 0.049.

### Clinical outcomes

Data on patients' weekly scores are presented in Table 2. All three cohorts of patients improved significantly over time, although no data were collected on what treatments they received. The mean change for depressed patients on the HAMD was 7.43 points (from 20.57 to 13.14), t(13) = 2.67, p = 0.019. The mean change for GAD patients on the HAMA was 10.06 points (from 14.63 to 4.56), t(15) = 4.48, p < 0.001, and the mean change for patients with panic disorder was 6.64 points on the PDDS (from 12.14 to 5.50), t(13) = 3.198, p = 0.007. Average time per assessment was 19.75 min for the HAMD, 21.43 min for the HAMA, and 17.45 min for the PDDS.

**Table 2:** Mean scores on HAMD, HAMA and PDDS by study visit

	Baseline	Week 1	Week 2	Week 3	Week 4	Week 5	Week 6
Depression (HAMD)	14.71	11.06	9.25	7.69	6.50	5.13	4.56
GAD (HAMA)	20.57	19.25	12.64	14.64	13.71	14.86	13.14
Panic disorder (PDSS)	12.14	7.14	5.36	5.29	5.00	6.21	5.50

### Patient and physician satisfaction

Satisfaction and comfort levels of the participating patients using telemedicine were very high (Table 3). A total of 94% indicated that they would be 'somewhat likely' or 'very likely' to participate again, and 86% said they would be 'somewhat likely' or 'very likely' to recommend to others participation in clinical studies using videoconferencing. Only one participant rated the experience 'somewhat negative', with the rest feeling either somewhat or very positive (91%) or neutral (7%). A total of 96% reported feeling comfortable talking to a person on a TV monitor, and 93% agreed that they were able to satisfactorily communicate their feelings to the interviewer. Although 38% somewhat or strongly agreed that they preferred to be interviewed by someone face-to-face, it is notable that 62% either did not agree, or neither agreed nor disagreed. Indeed, 89% agreed that they 'felt like [they were] talking to someone in the same room.' A total of 60% agreed that they would prefer to be interviewed in their doctor's office using this technology rather than have to travel to be interviewed by someone face-to-face.

**Table 3:** Patient satisfaction survey

	Very negative	Somewhat negative	Neutral	Somewhat positive	Very positive
1. How would you describe your overall experience with the study?	0 (0%)	1 (2%)	3 (7%)	17 (38%)	24 (53%)

	Very unlikely	Somewhat unlikely	Neither likely nor unlikely	Somewhat likely	Very likely

2. Would you be likely to recommend to a friend to participate in clinical studies using videoconferencing?	0 (0%)	1 (2%)	5 (11%)	10 (22%)	29 (65%)
3. Would you participate in another study using this technology?	1 (2%)	1 (2%)	1 (2%)	7 (16%)	35 (78%)

	Strongly disagree	Somewhat disagree	Neither agree nor disagree	Somewhat agree	Strongly agree

1. I was comfortable talking to a person on a TV monitor	0 (0%)	1 (2%)	1 (2%)	15 (33%)	28 (63%)
2. I was able to satisfactorily communicate my feelings to the interviewer	0 (0%)	2 (4%)	1 (2%)	8 (18%)	34 (76%)
3. Videoconferencing/teleconferencing is a good way to get a psychological evaluation	0 (0%)	1 (2%)	6 (13%)	12 (27%)	26 (58%)
4. The scheduling was flexible enough for me	0 (0%)	2 (5%)	1 (2%)	6 (13%)	36 (80%)
5. I was comfortable that the interviews were kept confidential	0 (0%)	1 (2%)	0 (0%)	8 (18%)	36 (80%)
6. I would prefer to be interviewed by someone face to face	3 (7%)	6 (13%)	19 (42%)	13 (29%)	4 (9%)
7. I established a good relationship with my interviewer	0 (0%)	1 (2%)	8 (18%)	19 (42%)	17 (38%)
8. I was able to see the interviewer satisfactorily	0 (0%)	1 (2%)	0 (0%)	9 (20%)	35 (78%)
9. I would prefer to be interviewed in my doctor's office using this technology than have to travel to be interviewed by someone face-to-face	2 (4%)	4 (9%)	12 (27%)	8 (18%)	19 (42%)
10. I was comfortable in the room alone	0 (0%)	1 (2%)	0 (0%)	7 (16%)	37 (82%)
11. It felt like I was talking to someone in the same room	0 (0%)	2 (4%)	3 (7%)	9 (20%)	31 (69%)
12. There were technical problems relating to the sound or image quality	33 (73%)	4 (9%)	4 (9%)	2 (4%)	2 (4%)
13. How good was the quality of the image?	0 (0%)	1 (2%)	2 (4%)	17 (38%)	25 (56%)
14. How good was the quality of the sound?	0 (0%)	0 (0%)	1 (2%)	13 (29%)	31 (69%)

	Never	Almost never	Occasionally	Often	Very often

15. Were the sessions interrupted by technical difficulties?	36 (82%)	5 (12%)	1 (2%)	1 (2%)	1 (2%)
16. How often do you use a computer?	6 (13%)	3 (7%)	11 (24%)	8 (18%)	17 (38%)
17. Before the study, how often did you use videoconferencing?	38 (85%)	2 (5%)	2 (5%)	2 (5%)	0 (0%)

Primary care physicians and staff had an average positive rating of 4.8 (out of 5) when asked the question, 'Would you be likely to participate in a telemedicine study again?' (Response options ranged from 1 (very unlikely) to 5 (very likely)).

## Discussion

Results of this study support the hypothesis that patients in primary care settings will enroll, participate in and complete research protocols using remote interviews conducted via videoconference. The waiting room screening process was time-efficient, accepted by patients, and resulted in an adequate yield of potential subjects. The 20% drop-out rate compares favorably to the drop-out rate found in clinical trials conducted at psychiatry sites [29,30]. A secondary positive outcome of this process is the identification of patients with mental disorders that may otherwise have gone undetected and untreated.

To facilitate patient participation in future studies with remote assessors, sites must foster a patient-friendly environment and ensure specific compensation for the site staff to align their incentives with that of the physician's practice. In addition, patient compensation may have played a factor in the completion rate. It is possible that the completion rate may have been less if patients received no compensation. The study process could have been improved by adding questions to the PHQ that screened out patients who did not meet other inclusion criteria (e.g., currently in treatment, etc.). For Site II enrollment, just such an amendment to the PHQ was provided and only a single patient who did not fit the inclusion/exclusion criteria made it through to the Phase II screening interview. Patient declines may also have been minimized by a shorter time window between screening and follow-up contact. Delay in scoring the PHQ and notifying patients that they qualified for the study may have resulted in lowering the participation rate. In the future, incorporating the informed consent process in the videoconference procedure may further enhance the ability to enroll patients. Dobscha and colleagues [31] successfully utilized this method of obtaining consent in a study of depressed primary care patients, and found patient satisfaction and acceptance was high, with no increase in patients lost to follow up.

In summary, the current study provides support for the use of primary care sites in clinical drug trials in psychiatry. Using primary care sites may help solve problems with patient recruitment, as well as overcoming some of the methodological problems associated with patients recruited in psychiatric settings. The use of independent raters has been shown to further improve clinical trial methodology by improving reliability and quality of assessments, decreasing bias, and, in a recent study, decreasing placebo response [32]. The combination of centralized independent raters and primary care settings should provide a powerful new approach to the conduct of clinical trials.

## Competing interests

JBWW, KAK and AE are employed by MedAvante, a company that provides centralized rating services for clinical trials.

## Authors' contributions

JBWW and AM conducted the clinical interviews, and helped with study design. AE helped with study design. KK conducted the statistical analyses and drafted the manuscript.
